# Comparative Evaluation of Accuracy, Completeness and Readability of Common Patient Queries Related to Prosthodontic Treatment by Two Artificial Intelligence Models (ChatGPT-4o and Gemini)

**DOI:** 10.7759/cureus.98458

**Published:** 2025-12-04

**Authors:** Litty Francis, Deepthi V S, Prasanth Viswambharan, Vivek V Nair

**Affiliations:** 1 Prosthodontics, Government Dental College, Thiruvananthapuram, IND

**Keywords:** artificial intelligence, dental implant, fixed partial denture, large language models, prosthodontics, removable partial denture

## Abstract

Introduction: Artificial intelligence (AI) is fundamentally characterized by the capacity of computer systems to execute tasks that traditionally require human intelligence through the application of sophisticated algorithms. The coming years are bound to witness an increase in the number of people relying on the AI language models for initial health-related queries. The reasons may include increased credibility of AI models, convenience and privacy, initial knowledge acquisition before their visit to a doctor and guidance regarding treatment options.

Aim: The aim of the study was to compare the performance of two commonly used AI models, Chat Generative Pretrained Transformer (ChatGPT-4o, OpenAI, San Francisco, United States) and Google Gemini Flash 2.5 (Google Bard, Google DeepMind, Mountain View, California, United States), in responding to common patient queries related to prosthodontic treatments and evaluate their responses in terms of accuracy, completeness, generation time, length and readability.

Materials and methods: In this study, 30 open-ended questions that are frequently asked by patients visiting prosthodontists were collected. Ten questions each from the domains (removable (partial and complete), fixed partial dentures and dental implantology) were submitted to both AI models: ChatGPT 4o and Google Gemini Flash 2.5. Four experts in the field of Prosthodontics with a minimum of 20 years’ experience, who were blinded to the study, evaluated the accuracy and completeness of the responses from the models. Accuracy, completeness, generation time, length and readability of the responses generated by the two AI models were compared using the Likert scale and Simple Measure of Gobbledygook (SMOG) Index.

Results: The comparison of outputs from two AI systems evaluated illustrated that Model B (Gemini) achieved 0.25 higher completeness and accuracy scores compared to Model A (ChatGPT). In terms of readability, Gemini generated outputs with a median SMOG index 1.46 points higher than ChatGPT (p = 0.004), suggesting a more advanced reading level.

Conclusion: Gemini demonstrated superior performance compared to ChatGPT, delivering more professional and technical content, while ChatGPT was more accessible and comprehensible for non-professionals and the general public.

## Introduction

Technology has become so deeply integrated into our daily lives that living without it now seems almost impossible. Intelligence is a quality we all admire and aspire to possess, and today, everything around us is becoming smart and intelligent. Artificial intelligence (AI) is fundamentally characterized by the capacity of computer systems to execute tasks that traditionally require human intelligence [1], through the application of sophisticated algorithms. Since its introduction at the Dartmouth conference [2], AI has significantly transformed the way we live. These systems analyze large volumes of data to identify patterns and solve problems based on the insights they gain from this learning. It now plays a significant role across various dental specialties, aiding in tasks such as radiographic interpretation through image analysis, caries detection, treatment planning, and more [3]. LLMs (large language models) are a specific type of advanced natural language processing (NLP) model, which are able to generate human-like text and perform complex language tasks such as text generation, question answering, creative writing and complex reasoning by learning from massive amounts of data [4]. The development of LLMs has made it much easier for both the general public and experts to find answers to even their most spontaneous or complex questions. The accuracy of answers provided by LLMs is dependent on the quantity, quality, and type of data used in their training. The internet is overflowing with information, and searching it for even the simplest queries has become a routine part of life. However, this overwhelming volume of information must be navigated carefully, as accurate and relevant details are often buried within a sea of content. This challenge affects everyone-professionals, students, and patients alike. An online survey about the health-related online activities showed that 57% first rely on the internet for researching the information related to a specific health condition or question with women (63%) more likely to do so [5].

ChatGPT [6] is a conversational AI system launched by OpenAI, an AI research and deployment company. A recent surge of studies could be seen to assess the abilities of AI models like ChatGPT to answer medically related questions. A study found that ChatGPT could serve as a significant tool providing information to the patients in oral and maxillofacial surgery (OMFS) [7]. There are several limitations, as ChatGPT is not connected to the internet, and it can occasionally produce incorrect answers, commonly referred to as hallucinations [8-10]. It has limited knowledge of the world and events after 2021 and may also occasionally produce harmful instructions or biased content [11]. On March 14, 2023, OpenAI introduced ChatGPT-4, an updated subscription-based model that performed better in comparison with the earlier versions. As the popularity of LLMs increased, other models like Bard (the predecessor of Gemini) were introduced by Google in 2023. In 2024, Bard was updated and relaunched as Gemini [12]. Microsoft launched the new Bing in February 2023. These LLMs can act as a Double-edged sword when the information provided by the user is insufficient and should be used with caution [13].

People rely on information provided on the internet to gain an understanding of their condition beforehand to safeguard themselves. Many studies have been conducted in the medical field to evaluate the accuracy of responses by the LLMs [14]. Researchers in dentistry have related studies in the field of oral and maxillofacial surgery [7], oral medicine [15], prosthodontics [16,17] and endodontics [18]. Additionally, the ability of chatbots to respond to questions in board-style dental knowledge has been assessed. Freire et al. [17] evaluated the accuracy and repeatability of answers generated by one AI chatbot (GPT-4) to clinicians’ procedural questions about removable partial dentures and tooth-supported fixed dental prostheses. AI models provide faster responses to queries and are user-friendly. The dental health-related facts, provided by the AI models, should be accurate, complete and comprehensible to all, to ensure their utility. There is no conclusive study as to which AI model is the best to answer the frequently asked questions by patients seeking prosthodontic treatment. ChatGPT and Gemini, the two commonly used AI models, were shown to perform with high accuracy consistently compared to newer AI models like Grok, Perplexity.ai, etc. in various medical specialties, from neuroradiology to physiology and clinical diagnosis. ChatGPT and Gemini generate structured, accurate and comprehensible responses as they have a large database compared to the emerging AI models. The aim of this study was to compare the performance of two commonly used AI models, Chat Generative Pretrained Transformer (ChatGPT-4o, OpenAI, San Francisco, United States) and Google Gemini Flash 2.5 (Earlier Google Bard, Google DeepMind, Mountain View, United States), in responding to common patient queries related to prosthodontic treatments. The model responses were evaluated in terms of accuracy, completeness, generation time, length and readability.

## Materials and methods

The study was initiated after obtaining Institutional Ethical Clearance (IEC No: DCT/IEC/SS/25/43). The two commonly used LLMs- ChatGPT-4o and Gemini Flash 2.5 were used for comparing the accuracy, completeness and readability of the given answers. In the first stage of this three-stage research, open-ended questions that are frequently asked by patients visiting prosthodontists were collected. The questions collected came from patient records and the clinical experience of various prosthodontists. The questions were categorized using specific domains into removable (partial and complete) dentures, fixed partial dentures, and dental implantology. Ten questions from each specific domain were included. The questions selected randomly, using the lottery method, from the collection of frequently asked questions (FAQs) were asked to the AI models, including information on prosthodontic treatment options like crowns & fixed partial dentures, removable partial & complete dentures, and implants.

In the next phase, questions were submitted to both AI models: ChatGPT (GPT-4o) and Google Gemini Flash 2.5. To standardize the methodology, the questions were submitted consecutively to both ChatGPT and Google Gemini Flash 2.5. Each question was submitted in a new window. The time to generate the responses to the query was recorded using a stopwatch, as well as total number of words for each response was noted. To ensure blinding of the evaluators, the identifiers of responses from AI models were removed; a set of 30 responses from Chat GPT was labelled as Model A, and a set of 30 responses from Gemini was labelled as Model B and sent for evaluation. Four experts in the field of Prosthodontics with a minimum of 20 years’ experience, who were blinded to the study, evaluated the accuracy and completeness of the responses from the models.

The evaluators assessed the response accuracy on a three-point Likert scale (Table 1).

**Table 1:** Rubric used to score the accuracy of the answers

	Experts’ grading	Description
1	Incorrect (0)	The answer provided is completely incorrect or unrelated to the question. It does not show an adequate understanding or knowledge of the topic
2	partially correct or Incomplete (1)	The answer shows some understanding or knowledge of the topic, but there are significant errors or missing elements. Although not entirely incorrect, the answer is not sufficiently accurate or complete to be considered appropriate.
3	Correct (2)	The answer is completely correct and shows a sound and accurate understanding of the topic.

Similarly, the completeness i.e. comprehensiveness and adequacy, of the responses were assessed on a three-point Likert scale (Table 2).

**Table 2:** Score used for the completeness of the answers

	Experts’ grading	Description
1	1 - Incomplete	addresses some aspects of the question, but significant parts are missing or incomplete;
2	2 - Adequate	addresses all aspects of the question and provides the minimum amount of information required to be considered complete;
3	3 - Comprehensive	addresses all aspects of the question and provides additional information.

The high score indicated more accurate and complete responses. The readability of the responses given was determined with the Simple Measure of Gobbledygook (SMOG) readability scores. The SMOG grade is a measure of readability developed by G. Harry McLaughlin in 1969 [19], recommended for use in healthcare. It was developed as an alternative to the Gunning fog index. The approximate conversion between the education level and SMOG readability score is given in Table 3.

**Table 3:** Approximate conversion between the education level and SMOG readability score SMOG: Simple Measure of Gobbledygook

Score	Education Level
4.9 or lower	Elementary school
5 - 8.9	Middle school
9 - 12.9	High school
13 - 16.9	Undergraduate
17 or higher	Graduate

Statistical analysis

The data collection was employed and maintained in Excel, and Stata version 16.0 (StataCorp, College Station, TX, USA) was used for analyzing the data. Descriptive statistics were expressed as frequency and percentage for categorical variables and median IQR for continuous variables. Normality of paired differences was assessed using the Shapiro-Wilk test. The Wilcoxon signed-rank test was employed to compare the median differences between the two AI models for completeness scores, accuracy scores, response time, readability scores, number of sentences, and number of syllables per output, since the distributions deviated from normality. Box plots were used to visualize the distribution of continuous variables across the two AI models. For categorical outcomes, including reading level and reading notes, marginal homogeneity was assessed using the Stuart-Maxwell test of symmetry. Inter-rater reliability was assessed using Cohen’s kappa statistic to evaluate the level of agreement between raters. A p-value < 0.05 is considered statistically significant.

## Results

**Figure 1:**
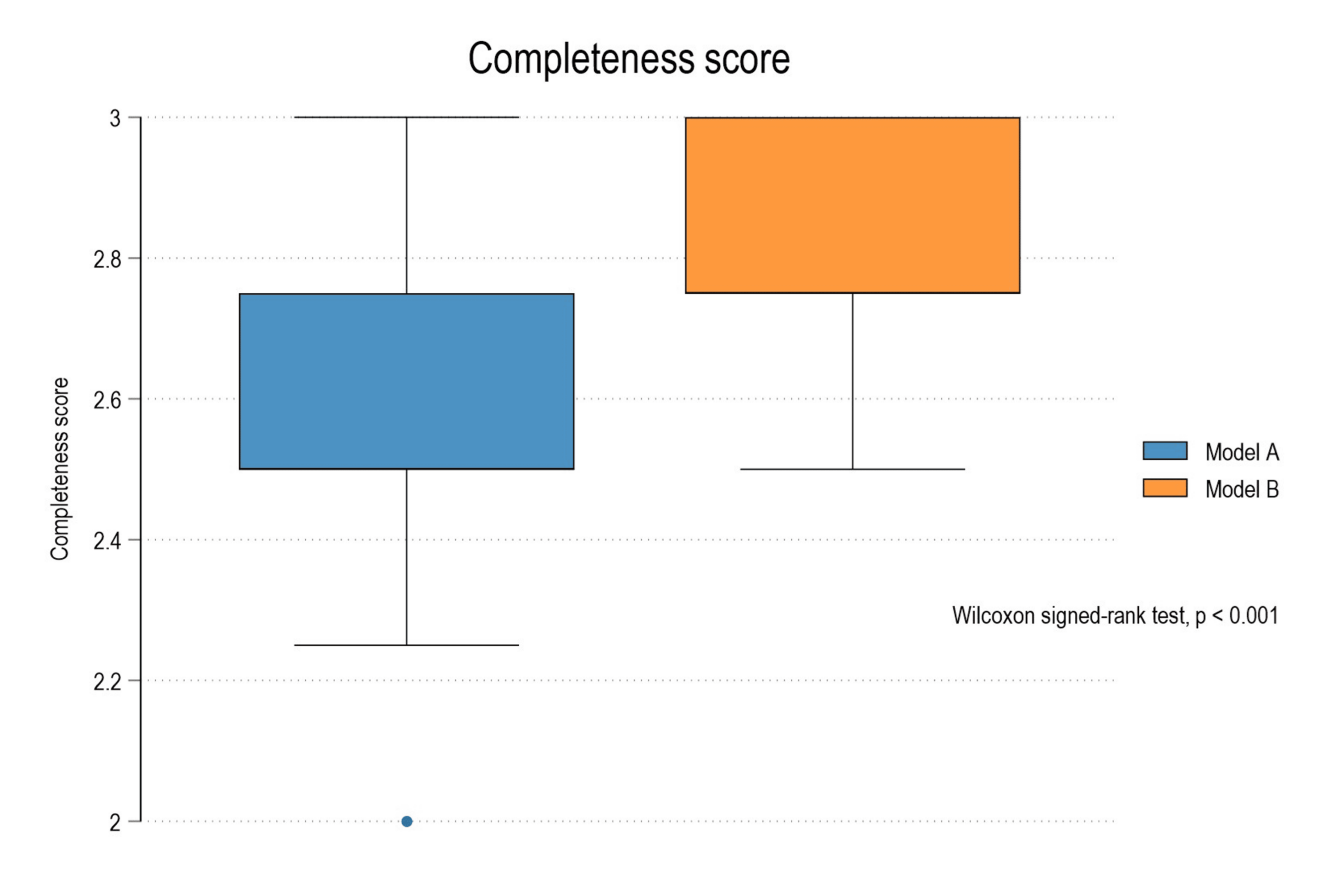
Box plot diagram depicting comparison of completeness between Model A and Model B

**Figure 2:**
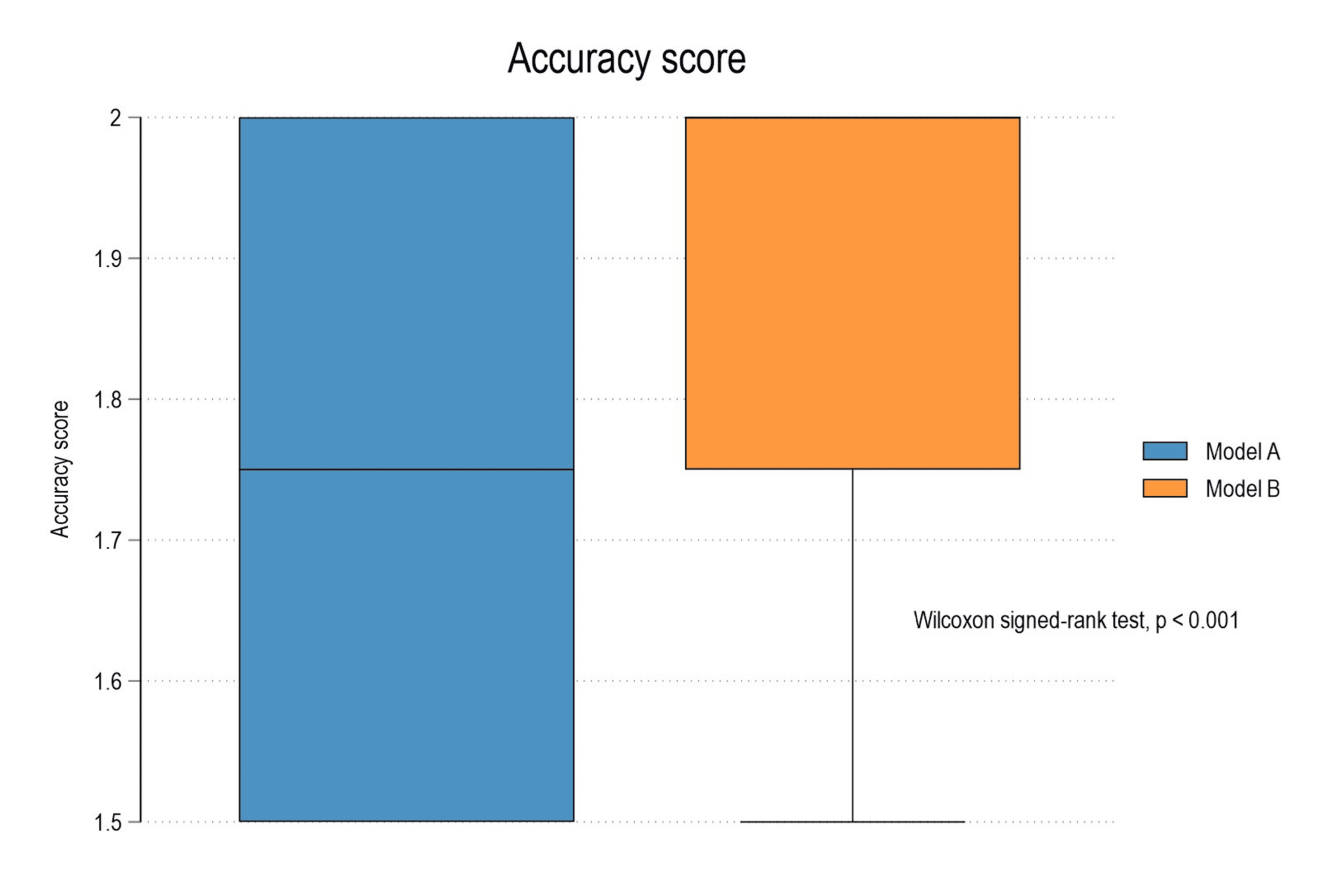
Box plot diagram depicting comparison of accuracy between Model A and Model B

The comparison of outputs from two AI systems evaluated using 30 prescribed medical prompts related to prosthodontics was conducted. The results illustrate that Model B (Gemini) achieved 0.25 higher completeness and accuracy scores compared to Model A (ChatGPT), with the Wilcoxon signed-rank test confirming the differences as statistically significant (p < 0.001 for both) (Table 4; Figures 1, 2).

**Table 4:** Comparison of the outputs of Model A(ChatGPT) and Model B (Google Gemini) Accuracy and completeness scores were compared using the Wilcoxon signed-rank test, confirming a statistically significant difference. For categorical outcomes like reading level and reading notes, marginal homogeneity was assessed using the Stuart–Maxwell test of symmetry.

Variables	Model A	Model B	Difference (B-A)	Value of statistic (t-value, z-value or chi^2-value)	DF	p-value
Completeness (Score range 0-3)	2.50 (2.50, 2.50)	2.75 (2.75, 3.00)	0.25	-4.28	29	<0.001
Accuracy (Score range 0-2)	1.75 (1.50, 2.00)	2.00 (1.75, 2.00)	0.25	-3.49	29	<0.001
Response time (in seconds)	15.55 (14.10, 18.40)	8.05 (7.40, 9.10)	-7.5	4.73	29	<0.001
Reading score	12.23 (10.98, 13.43)	13.69 (12.71, 14.49)	1.46	-2.81	29	0.004
Sentence	11.00 (9.00, 12.00)	22.5 (20.00, 27.00)	11.5	8.83	29	<0.001
Syllables	284.5 (242.00, 344.00)	777 (670.00, 929.00)	492.5	13.77	29	<0.001
Reading Level	Model A	Model B	Value of statistic (t-value, z-value or chi^2-value)		DF	P-value
7th Grade	18 (60.00)	8 (26.67)	13		2	0.001
Under graduate	9 (30.00)	22 (73.33)				
Graduate	3 (10.00)	0 (0.00)				
Reading note	Model A	Model B	Value of statistic (t-value, z-value or chi^2-value)		DF	P-value
Plain English	18 (60.00)	8 (26.67)	13		2	0.001
Difficult to read	9 (30.00)	22 (73.33)				
Very difficult to read	3 (10.00)	0 (0.00)				

**Figure 3:**
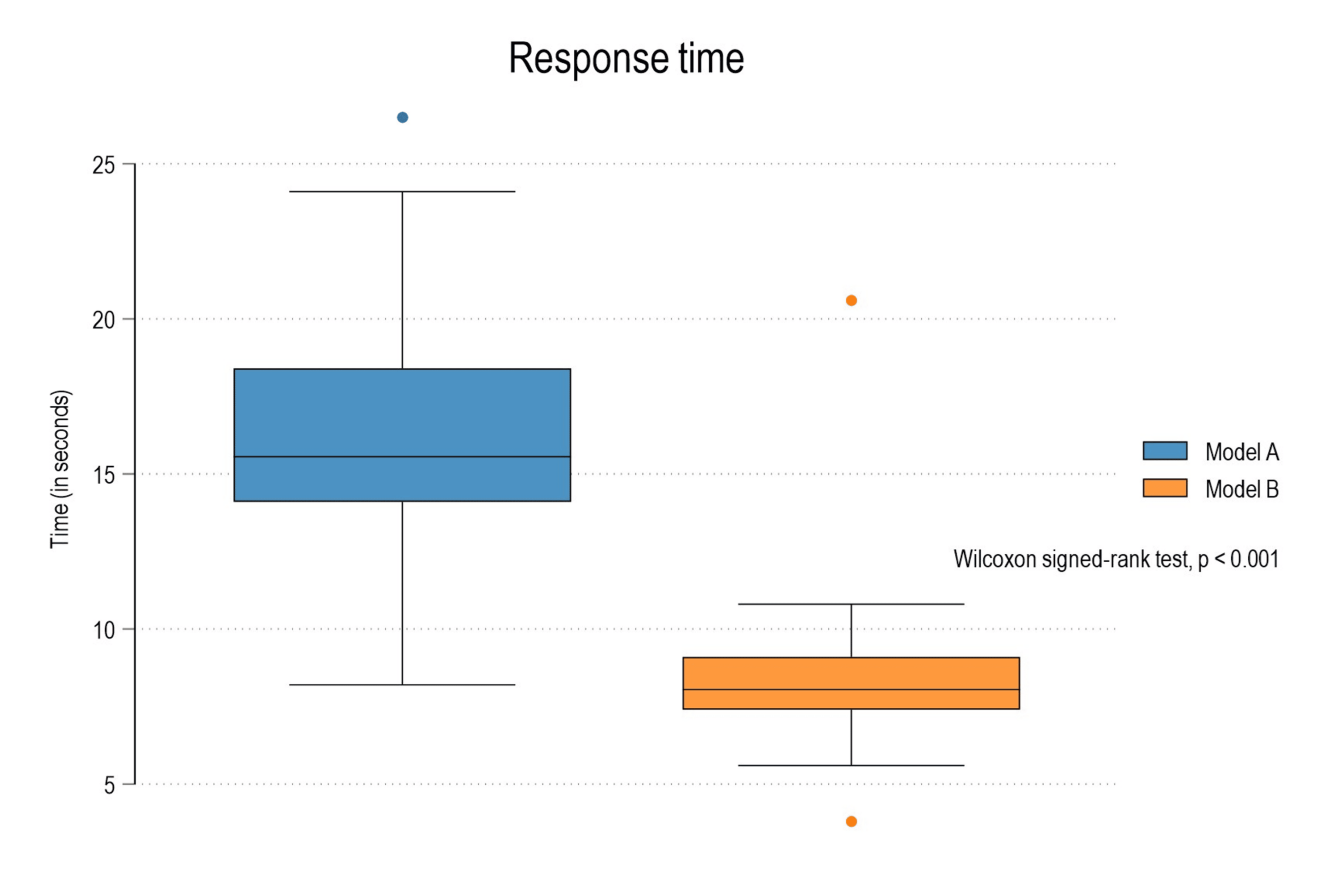
Box plot diagram depicting comparison of response time between Model A and Model B

Model B also responded significantly faster, with a median response time 7.5 seconds shorter than Model A, approximately half of Model A’s response time (Table 4; Figure 3)

**Figure 4:**
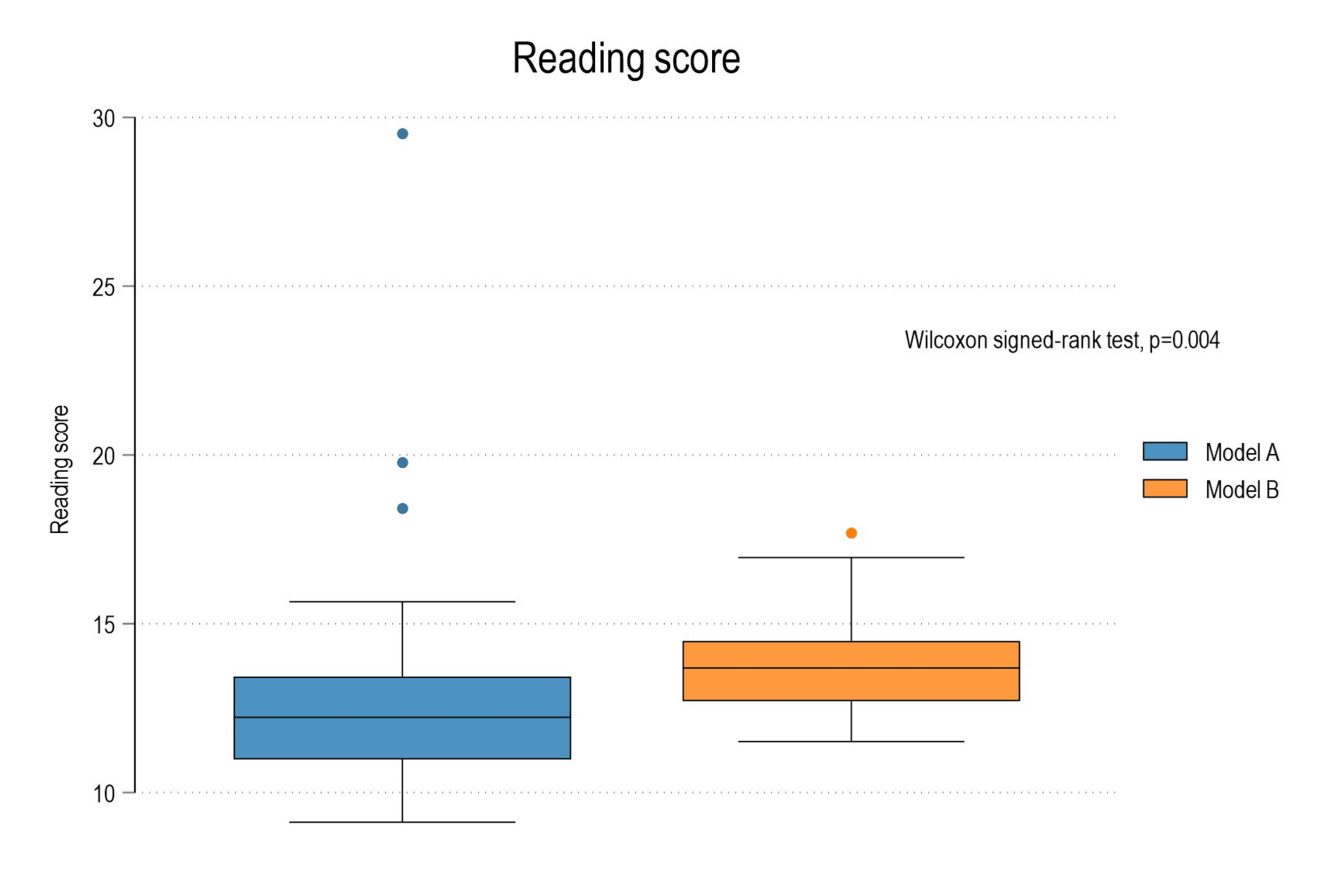
Box plot diagram depicting comparison of the reading score between Model A and Model B

In terms of readability, Model B generated outputs with a median SMOG index 1.46 points higher than Model A (p = 0.004), suggesting a more advanced reading level. Furthermore, Model B produced more than twice the number of sentences and syllables compared to Model A, with these differences also statistically significant (p < 0.001) (Table 4; Figure 4).

The analysis of reading notes and reading level shows that Model A predominantly generated outputs at the 7th-grade level with 60% in plain language, while Model B produced outputs at the undergraduate level, with 73% categorized as difficult language.

As shown in Table 5, the model A accuracy score suggests that the raters had fair agreement, with a percentage agreement of 56% (95% CI: 45-66%, p < 0.001). The completeness score indicates poor-to-fair agreement, with a percent agreement of 43% (95% CI: 38-48%, p < 0.001). The accuracy score for Model B indicates that there was high agreement among raters, with a percent agreement of 80% (95% CI: 69-90%, p < 0.001), and the completeness score yielded a percent agreement of 72% (95% CI: 62-82%, p < 0.001), corresponding to moderate-to-substantial agreement.

**Table 5:** Inter-rater agreement Cohen’s kappa statistic was used to assess Inter-rater reliability

	Kappa coefficient	SE	t-value	p-value
Accuracy Score				
Model A	0.56 (0.45 - 0.66)	0.051	11.04	<0.001
Model B	0.80 (0.69 - 0.90)	0.049	16.16	<0.001
Completeness Score				
Model A	0.43 (0.38 - 0.48)	0.026	16.6	<0.001
Model B	0.72 (0.62 - 0.82)	0.048	14.79	<0.001

In short, the inter-rater agreement was consistently higher for Model B compared to Model A, for both accuracy and completeness scores. Model B achieved moderate-to-substantial agreement (72-80%), but Model A only reached poor-to-fair agreement (43-56%). The above findings suggest that Model B provided more consistent and reliable outputs across the perspectives of the four raters compared with Model A.

## Discussion

The use of the internet by the public for health-related information is on the rise and may vary with age and socio-economic status [20]. Compared to the earlier versions of LLMs from Google and OpenAI, the newer ones demonstrate substantial improvements. Google's Gemini Advanced has been introduced as a strong competitor to OpenAI’s GPT-4. The difference between the LLMs depends mainly on their training data set. GPT-4o is the default model in the free version of ChatGPT. Relying on AI models to access information beforehand is now a widespread trend, and the purpose of the study was to compare the accuracy, completeness, and readability of two AI model responses to common patient queries relating to prosthodontics.

In this study, the accuracy of the answers provided by the Gemini was significantly greater than the OpenAI ChatGPT model. This is in total contrast to the findings in a study that compared 4 different LLMs [16]. In this, the Chat GPT model gave more accurate responses compared to other models like Bing and Gemini. Another study done to assess the performance of three LLMs (GPT-3.5, GPT-4, and Google Bard) on a question bank designed specifically for neurosurgery oral boards examination preparation also showed greater accuracy for the ChatGPT model than the AI model Gemini [21]. According to a study to evaluate and compare the accuracy and consistency of responses generated by publicly available ChatGPT-3.5 and Google Bard to non-expert questions related to lung cancer prevention, screening, and terminology commonly used in radiology reports, ChatGPT-3.5 was shown to provide correct or partially correct answers than Google Bard (Gemini), approximately by 1.5-fold [22].

The answers provided by Gemini achieved 0.25 higher completeness when compared to the ChatGPT model and are statistically significant. In the present study, Google Gemini provided both accurate and complete responses when compared to ChatGPT, which contradicts the findings of the study conducted by Daraqel et al. [23], where ChatGPT responses were marginally more accurate than Google Gemini (Bard). According to a study by AlSagri et al., to assess the capabilities of ChatGPT-3.5 and Gemini as scientific assistants [24], Gemini was more accurate than ChatGPT-3.5.

The overall performance of the AI models is not only based on the accuracy, completeness, and reliability of the information but also better user experience, data protection, creative interaction with the user [25], etc. User experience is largely influenced by both the time taken to generate a response and length of the response. In today’s fast-paced world, people expect quick results and are unwilling to wait. Likewise, patients prefer information that is concise and delivered within a short timeframe. In this aspect, the Google Gemini has a definite advantage over the ChatGPT model, as the response generation time was significantly faster, with a median response time 7.5 seconds shorter than ChatGPT-approximately half of ChatGPT's response time. This is consistent with the findings of studies by Daraqel et al. [23], where Google Bard (Gemini) had an average response time which was 10 seconds faster for each question compared to Chat GPT, and Patil et al. [26] where a mean generation time per response of 7.55 seconds with Google Bard (Gemini) compared to 26.79 seconds with ChatGPT while responding to radiology board examination questions. Although Gemini answered the questions faster, its response was verbose. A similar observation was made by Patil et al. [26] in their study. Gemini is built to access data from the internet in real time and to quickly find and incorporate the most current information into its answers, leading to a faster response time for factual questions.

The recommended reading levels are put forward by various agencies like Agency for Healthcare Research and Quality (AHRQ) (5th to 6th grade) [27], the National Institute of Health (7th to 8th grade), the American Medical Association (6th grade) and the UK’s National Health Service (11-14 years old) [28] for better understanding of the disseminated information by the public. Our study showed that both ChatGPT and Google Gemini generated responses that were difficult to comprehend for the common man. Google Gemini generated outputs with a median SMOG index 1.46 points higher than ChatGPT, suggesting a more advanced reading level. ChatGPT predominantly generated outputs at the 7th-grade level, with 60% in plain language, while Gemini produced outputs at the undergraduate level, with 73% categorized as difficult language. Patient educational materials should be accurate, readable, and of high quality. The health information intended for educating the patients should be accurate, complete, and in a simple, non-confusing, readable language, as this may influence the patients’ decisions as well as overall participation in the treatment and care provided [29]. AI model responses obtained were consistently difficult to comprehend, regardless of their accuracy. The readability and accuracy of the responses may be improved by providing an accurate and complete dataset for training of the AI models, ensuring valid health information [20].

However, there are some limitations to this study. While Gemini and ChatGPT are the commonly used LLMs, newer alternatives are constantly emerging. In this study, only the two leading AI models were used, and the numerous other models like Perplexity.ai, Grok, Microsoft copilot, Claude, etc. were not included. Ten questions from each section may not sufficiently cover the entire specialty-related patient queries. The limited sample size of 30 questions restricts the generalizability of the findings, particularly considering the vast range of patient concerns encountered in prosthodontics. The study is also limited by the fact that the comparison of responses was performed only between two different AI models. These were not compared with human-generated responses, which would be more specific, empathetic and clear, tailoring the response to individual contexts. Another limitation of the study is the exclusion of the patient perspective, resulting in conclusions about comprehension that are based exclusively on readability scores instead of direct evaluation of real-world patient experience.

The future studies can include the newer AI models to find the most accurate and comprehensible model for disseminating health-related information to a diverse population. Studies comparing the AI-generated and human-generated responses may help in building a more accurate and complete health-related information database. There is a scope for further research in evaluating the patient's interpretation of the health-related information, their understanding of the material, and patient satisfaction. Explainable AI may as well be integrated with AI models in future research, as it accounts for transparency and ensures reasonable decision-making.

## Conclusions

The coming years are bound to witness an increase in the number of people relying on the AI language models for initial health-related queries. The reasons may include increased credibility of AI models, convenience and privacy, initial knowledge acquisition before their visit to a doctor and guidance regarding treatment options. The dataset to which an LLM has access needs to be comprehensive, up-to-date, and vetted to ensure that the latest scientific findings are included and avoid bias from sources that contain misinformation or disinformation. Even though the present LLMs are not perfect as patient education providers, AI tools can be trained to provide health educational information. This reiterates the need for collaboration between the AI model developers and the healthcare providers in developing accurate and complete health-related information. In this study, Google Gemini demonstrated superior performance compared to ChatGPT, delivering more professional and technical content, while ChatGPT was more accessible and comprehensible for non-professionals and the general public. ChatGPT provided more natural, humanlike responses, making it a user-friendly model.

## Appendices

**Table 6:** Commonly asked questions by the patients visiting prosthodontists submitted to the AI models ChatGPT and Gemini The questions were categorized using specific domains into fixed partial dentures, removable (partial and complete) dentures and dental implants

Fixed Partial Denture (Crown and Bridge)	
1	What happens if I don’t get a dental bridge?
2	How long does a dental bridge last?
3	Can I eat normally with a crown?
4	How long does it take to get a dental bridge?
5	Is there a possibility of swallowing my dental bridge?
6	Will you anesthetize before preparing for a dental bridge?
7	I have a root canal treated tooth; is it necessary to place a cap over the tooth?
8	Which specialist should I see for replacing my tooth?
9	Which treatment option is aesthetical for replacing front teeth?
10	What happens if I do not replace my lost tooth?
Removable Dentures (Complete and Partial)	
11	Can I have all types of food using my complete dentures?
12	Are removable partial dentures comfortable?
13	Is taking a dental impression painful?
14	Can I eat normally with complete dentures?
15	How long do complete dentures last?
16	Can I wear my complete dentures all the time?
17	Can I use regular toothpaste to clean my partial dentures?
18	How do I care for my removable partial dentures?
19	What happens if my partial denture breaks?
20	Will removable partial dentures affect my speech?
Dental Implants	
21	What is the success rate of dental implants?
22	How do I care for my dental implants?
23	Are dental implants better than dentures or bridges?
24	How much do dental implants cost?
25	Is the procedure for dental implant placement painful?
26	What are the benefits of dental implants over other tooth replacement options?
27	Can I eat normally after getting dental implants?
28	Do I need to take medications for a dental implant treatment?
29	Do I need to take a radiograph before placing a dental implant?
30	Is there any side effect for dental implant treatment?
